# The microbial community associated with *Parascaris* spp. infecting juvenile horses

**DOI:** 10.1186/s13071-022-05533-y

**Published:** 2022-11-04

**Authors:** Jennifer L. Cain, Jamie K. Norris, Nichol E. Ripley, Parul Suri, Constance A. Finnerty, Holli S. Gravatte, Martin K. Nielsen

**Affiliations:** Maxwell H. Gluck Equine Research Center, University of Kentucky, Lexington, KY USA

**Keywords:** Parasite microbiota, Nematode, Parasite, Ascarid, *Parascaris*, Equine parasite

## Abstract

**Background:**

Parasitic nematodes, including large roundworms colloquially known as ascarids, affect the health and well-being of livestock animals worldwide. The equine ascarids, *Parascaris* spp., are important parasites of juvenile horses and the first ascarids to develop widespread anthelmintic resistance. The microbiota has been shown to be an important factor in the fitness of many organisms, including parasitic nematodes, where endosymbiotic *Wolbachia* have been exploited for treatment of filariasis in humans.

**Methods:**

This study used short-read 16S rRNA sequences and Illumina sequencing to characterize and compare microbiota of whole worm small intestinal stages and microbiota of male and female intestines and gonads. Diversity metrics including alpha and beta diversity, and the differential abundance analyses DESeq2, ANCOM-BC, corncob, and metagenomeSeq were used for comparisons.

**Results:**

Alpha and beta diversity of whole worm microbiota did not differ significantly between groups, but Simpson alpha diversity was significantly different between female intestine (FI) and male gonad (MG) (*P*= 0.0018), and Shannon alpha diversity was significantly different between female and male gonads (*P* = 0.0130), FI and horse jejunum (HJ) (*P* = 0.0383), and FI and MG (*P*= 0.0001). Beta diversity (Fig. 2B) was significantly different between female and male gonads (*P* = 0.0006), male intestine (MI) and FG (*P* = 0.0093), and MG and FI (*P* = 0.0041). When comparing organs, *Veillonella* was differentially abundant for DESeq2 and ANCOM-BC (*p* < 0.0001), corncob (*P* = 0.0008), and metagenomeSeq (*P* = 0.0118), and *Sarcina* was differentially abundant across four methods (*P* < 0.0001). Finally, the microbiota of all individual *Parascaris* spp. specimens were compared to establish shared microbiota between groups.

**Conclusions:**

Overall, this study provided important information regarding the *Parascaris* spp. microbiota and provides a first step towards determining whether the microbiota may be a viable target for future parasite control options.

**Graphical abstract:**

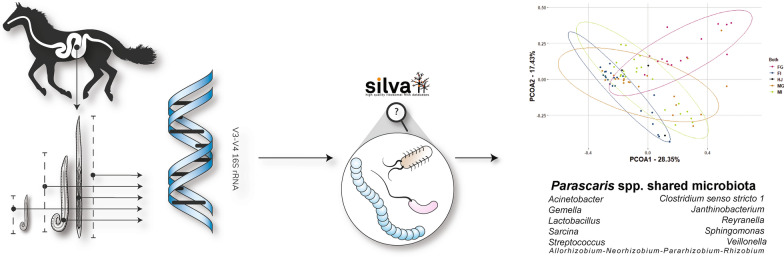

**Supplementary Information:**

The online version contains supplementary material available at 10.1186/s13071-022-05533-y.

## Background

The order Ascaridida consists of nematode parasites colloquially known as roundworms that infect a variety of host species and include important parasites of poultry, fish, dogs, cats, cattle, weasels, bears, pigs, horses, and humans. Ascarids can cause a wide array of clinical signs that can result in severe consequences, including death, which causes large economic losses in the agriculture industry [1–5]. The equine ascarids, *Parascaris* spp., are considered by veterinary parasitologists to be the most pathogenic parasites affecting juvenile horses worldwide [6, 7]. These parasites can cause coughing, lethargy, poor appetite, diarrhea, nasal discharge [6, 7], liver and lung lesions [8, 9], and impaction colic that may require surgical intervention [7, 10–12].

At present, *Parascaris* spp. are the only ascarid parasites that have evolved widespread anthelmintic resistance. Anthelmintic resistance has been reported in all three drug classes available for the treatment of *Parascaris* spp. in horses—macrocyclic lactones [13–38], tetrahydropyrimidines [13, 31–33, 39, 40], and benzimidazoles [13, 33, 42]—in multiple countries and continents. Anthelmintic resistance is of major concern in veterinary parasitology. In livestock parasitology, anthelmintic resistance is widespread in many important parasite species [43, 44] and has been for decades. It is also a rising concern in companion animal parasitology [45]. It is thought that frequent anthelmintic treatment intervals have contributed to the development of resistance and thus necessitate treatment efficacy monitoring programs [46], and some parasitologists believe that medical parasitology needs to learn from veterinary parasitology by reducing treatment frequency, identifying causes of resistance, and monitoring for decreased treatment efficacy [46–50]. Little has changed, however, in public health approaches to parasite control [50], and with the lack of new drug development, it is imperative to explore alternative options for parasite control as anthelmintic resistance becomes a major concern in other ascarid species, and before current drugs completely stop working against *Parascaris* spp.

Over the past couple of decades, the microbiota has been associated with a plethora of health outcomes including but not limited to Crohn’s disease [51], cancer [52], depression [53], equine oral health [54], and bovine respiratory disease [55]. In recent years, microbiota manipulation has emerged as a method to achieve a variety of results, including improved health outcomes in medicine, and is being explored in veterinary science [56–58], coral reefs [59, 60], bioremediation [61], and agriculture [62]. One particularly powerful example of the practical application of microbiota manipulation is that of endosymbionts in the genus *Wolbachia* which are found in filarial nematodes. *Wolbachia* are important for female worm development [63], larval and microfilarial development, molting, survival [64–66], and embryogenesis [67, 68]. Antibiotics in combination with anthelmintics [69, 70] were initially used as a treatment for filarial nematodes in humans, and two candidate drugs were recently developed and are in clinical trials for the treatment and prevention of filariasis [71, 72].

Despite the successful use of the microbiota for control of filarial nematodes, parasite microbiota as a whole remain understudied. Four microbiota studies have been completed for *Haemonchus contortus*, the most important nematode parasite affecting small ruminants, and have explored the microbiota of life stages [73, 74] and sexes [73, 75], the effect of antibiotics on parasite longevity [76], and the identification of intracellular bacteria via microscopy [74]. Two microbiome studies have been completed for *Trichuris* spp., an important parasite of humans and pigs [77, 78]. Microbiota studies have also been completed for *Schistocephalus solidus* [79], *Coitocaecum parvum* [80], and *Philophthalmus attenuatus* [81], all of which are flatworms infecting fish. The microbiota has also been associated with various factors in nematodes affecting plants, including virulence [82], life stages [83], development [84], and host symbiosis [85, 86], in *Bursaphelenchus* spp., endosymbiosis in *Xiphinema americanum* [87], *Radopholus similis* [88], and *Heterodera glycines* [87], and acquisition of parasitism genes in *Meloidogyne* spp. [90, 91].

In 2019, a paper was published highlighting 100 questions that need to be addressed in livestock helminthology research, voted on from 385 questions submitted by veterinary parasitology researchers in an effort to help close knowledge gaps and direct research efforts; the nematode microbiome was number 73 on that list [92]. Subsequently, a few papers have been published calling for more research and highlighting potential challenges, reinforcing the notion that helminth microbiota research is an important up-and-coming area of study [93–96]. This study aimed to, for the first time, characterize the bacterial population within *Parascaris* spp. by determining a shared microbiota and comparing microbiota diversity metrics for the whole worm at different life stages. Additionally, *Parascaris* spp. provided a unique opportunity to delve further into the parasite microbiome by studying individual organs such as the gonads and intestine due to its large size, which makes it easy to dissect, and allows for comparisons within the worm itself, which has not previously been done for helminth microbiome studies.

## Methods

### Parasites

All parasites were collected between August 2019 and November 2020 from 5-month-old foals euthanized as part of a routine research program under the University of Kentucky Institutional Animal Care and Use Committee (IACUC) protocol 2012-1046. Foals were not weaned prior to euthanasia and lived outdoors on pasture within the same herd 24/7, with free access to hay and daily grain. Intestinal content samples from the jejunum were also collected at necropsy. Parasites were placed into phosphate-buffered saline (PBS) immediately after removal from the small intestine, rinsed with water, and then placed into sterile PBS for further processing. Jejunal samples were snap-frozen in liquid nitrogen and stored in an −80 °C freezer. For the whole worm microbiota, three adult male, three adult female, and three immature worms were collected from each of three foals. Parasites were serially washed in 70% ethanol and sterile water three times before being snap-frozen in liquid nitrogen and stored in an −80 °C freezer until DNA extraction. Once thawed, a section from the center of adult parasites, determined by folding the worm in half and taking an approximately 2.5 cm section, was used for DNA extraction. Whole immature parasites were used for DNA extraction due to their smaller size. For the organ microbiota, a total of 46 adult *Parascaris* spp. (24 male and 22 female) were collected from three foals. The parasite surface was washed with 70% ethanol, and the worm was dissected using individually packaged sterile scalpels. All other tools were sterilized with 70% ethanol. Gonads and intestines were dissected from each individual parasite and placed into sterile 15 ml tubes, snap-frozen in liquid nitrogen, and stored in an −80 °C freezer.

### DNA extraction

DNA extraction was completed using the Zymo Quick-DNA Fecal/Soil Microbe Kit (Zymo Research Corporation, Irvine, CA, USA) with the following modifications. First, samples were placed in a 2 ml MP Biomedicals (Santa Ana, CA, USA) Lysing Matrix A tube with 750 µl of BashingBead™ buffer and then placed in a Bead Ruptor 12 (Omni International, Inc., Kennesaw, GA, USA) for two 90-s rounds on high. Final elution was performed using 75 µl of 10 mM Tris–HCl buffer (pH 8.5; bioWORLD, Dublin, OH, USA). DNA quantification was performed at the University of Kentucky Genomics Core Laboratory using the Qubit 2.0 (Thermo Fisher Scientific, Waltham, MA, USA).

### Next-generation sequencing (NGS) library preparation and sequencing

Library preparation for gonad, intestine, and whole worm samples was completed using the Illumina 16S metagenomic sequencing protocol (Illumina, San Diego, CA, USA, 2013). Female gonad samples were additionally prepared using the Swift Amplicon™ 16S+ITS Panel (Integrated DNA Technologies, Coralville, IA, USA) following the manufacturer’s instructions. Quantification was performed on an Agilent Technologies Stratagene Mx3000P (Santa Clara, CA, USA) using the Collibri^™^ Library Quantification Kit (Invitrogen, Waltham, MA, USA), following the manufacturer’s instructions, and quality analysis was performed using the Agilent 2100 Bioanalyzer (Agilent Technologies, Santa Clara, CA, USA). Library pooling was completed following the respective protocols, and sequencing was performed with the Illumina MiSeq^™^ (San Diego, CA, USA) using the MiSeq™ reagent kit v3 2 × 300 (Illumina) at the University of Kentucky Genomics Core Laboratory.

### Negative controls

Three negative reagent controls were maintained throughout the process, from DNA extraction to sequencing. All amplicon sequence variants (ASVs) found in these negative reagent controls were subsequently removed from all downstream analysis.

### Sequence processing

Raw paired 16S amplicon sequence data was converted into and retrieved as fastq files from the Illumina BaseSpace (https://basespace.illumina.com) interface using an Apple Mac Pro (Apple, Inc., Cupertino, CA, USA) running macOS High Sierra 10.13.6. Unless otherwise noted, default settings were used. Fastq quality was assessed, and adapter sequences and low-quality reads were removed using dada2 (version 1.22.0) [97]. A conservative minimum read length of 250 nucleotides was imposed for all reads. R (4.1.2) [98], BiocManager (1.30.16) [99] and Bioconductor libraries BiocStyle (2.22.0) [100], phyloseq (1.38.0) [101], DECIPHER (2.22.0) [102], phangorn (2.8.1) [103], decontam (1.140) [104], and ggplot2 (3.3.5) [105], as well as standard R libraries gridExtra (2.3) [106] and knitr (1.37) [107], were used in amplicon sequence analysis. The decontam package removed common contaminants in the data using control sample and DNA quantification data. The plotQualityProfile function provided by the dada2 R package was used to visualize a summary of the distribution of quality scores for a selection of forward and reverse reads and to assess quality thresholds. The function filterAndTrim was used to filter paired reads. In order to reduce computation time by reducing redundant comparisons, the derepFastq function was used to dereplicate amplicon sequences contained within the filtered data, producing a series of “unique sequences” with corresponding “abundance” estimates. Error rates were estimated using the learnErrors function and plotted using the plotErrors function to assess whether error rates were reasonably well estimated. Samples were clustered and denoised using the dada function, reducing sample error and inferring membership composition of the samples. Paired reads were merged and tabularized, and chimeric sequences were removed using the mergePairs, makeSequenceTable, and removeBimeraDenovo functions. Phyloseq and the SILVA non-redundant rRNA sequence library (v132) [108, 109] were used to analyze microbiota data and assign taxonomic rankings. All ASVs found in control samples, identified as Eukaryota, or without a named phylum were removed from downstream analysis.

### Statistical analysis

Prior to conducing diversity analyses, all abundance counts were converted to relative abundance by aggregating ASV data to genus and then dividing genus abundance counts by total reads for a particular sample. Diversity analysis was conducted on relative abundance data using the vegan (2.5–7) [110] package for R. Alpha diversity was calculated using both the Shannon and Simpson diversity indexes. Alpha diversity and relative abundance of individual genera were tested at the genus level for normality using the Shapiro–Wilk test. All normal distributions were then tested for statistical significance using analysis of variance (ANOVA) with Tukey adjustment, and all nonparametric distributions were tested using the Kruskal–Wallis and Dunn tests with Bonferroni correction. Beta diversity was calculated using the Bray–Curtis dissimilarity and was visualized using principal coordinate analysis. Statistical significance was tested for beta diversity using a beta-diversion calculation, which was then tested for significance using ANOVA with Tukey correction using the hagis (3.1.3) [111] package in R. Shared microbiota were determined using a value of > 0.5% relative abundance in > 20% of the samples. Analyses were completed using the phyloseq, knitr, microbiome (1.16.0) [112], dplyr (1.0.8) [113], lsmeans (2.30–0) [114], FSA (0.9.3) [115], and ape (5.6–2) [116] packages in R. Statistical analyses were conducted with α = 0.05.

### Differential abundance analysis

Differential abundance analysis was completed using four different methods that were then compared to determine commonalities in differentially abundant taxa. This analysis was completed in R using the tidyverse (1.3.1) [117], phyloseq, edgeR (3.36.0) [118], DEFormats (1.22.0) [119], DESeq2 (1.34.0) [120], apeglm (1.16.0) [121], corncob (0.2.0) [122], ANCOMBC (1.4.0) [123], eulerr (6.1.1) [124], and metagenomeSeq (1.36.0) [125] libraries. The four differential abundance analysis methods used were ANCOM-BC, DESeq2, corncob, and metagenomeSeq, and results were compared with a Venn diagram using eulerr. Four methods were chosen and compared in order to encompass different statistical methods.

## Results

### Sequencing results

The Illumina MiSeq^™^ sequencing run included whole worm, gonad, and intestinal specimens in addition to three reagent control for a total of 129 samples and yielded 11.24 gigabases (Gb) with 87.57% passing filter. After processing through the decontam run, 7,785,001 reads remained with a mean of 60,349 reads per sample (range: 45–308,386) and a total of 4635 ASVs. There were 77 ASVs found in negative controls, which were removed from all samples for subsequent analysis. The taxonomic assignment rate after negative controls were removed was as follows: 98.95% kingdom; 80.30% phylum; 79.88% class; 79.25% order; 75.93% family; and 64.98% genus.

Whole worm: After final sequence processing, a total of 132,375 reads remained for 31 whole worm microbiota samples, with a mean of 4270 reads per sample (range: 225–22,940). Prior to downstream analysis, all samples with less than 1000 reads per sample were removed [80, 81], along with the single sample of isolated eggs, leaving 26 samples (3 horse, 7 male, 8 female, 8 immature) for diversity and differential abundance analysis.

Organs: After final sequence processing, a total of 292,667 reads remained for 95 intestinal and gonad samples, with a mean of 3080 reads per sample (range: 0–11,148). Prior to downstream analysis, all samples with less than 200 reads were removed in order to maintain sample sizes between groups, leaving 83 samples (3 horse jejunum [HJ], 20 male gonad [MG], 23 male intestine [MI], 15 female gonad [FG], 22 female intestine [FI]) for diversity and differential abundance analysis.

### Whole worm microbiota of small intestinal stages of *Parascaris* spp.

**Fig. 1:**
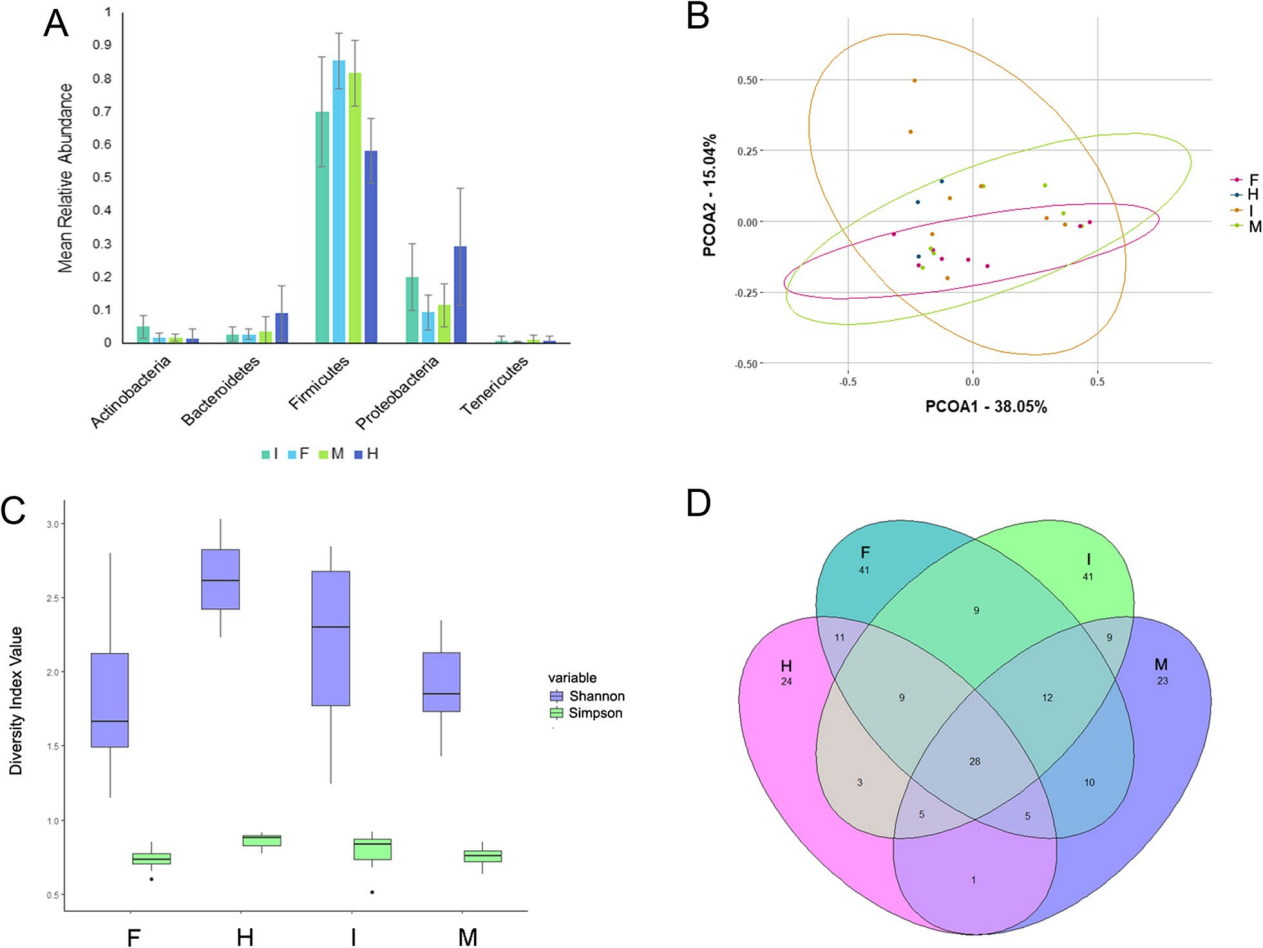
Diversity metrics, phylum relative abundance (RA), and shared genera for whole worm microbiota. **A** Mean RA of the five most abundant phyla, where error bars represent 95% confidence intervals. **B** Principal coordinate analysis plot based on Bray–Curtis dissimilarities. **C** Alpha diversity box plot showing both Shannon and Simpson alpha diversity, where • denotes outliers. **D** Venn diagram showing number of shared genera between groups. *F* female, *M* male, *I* immature, *H* horse

Overall, a total of 22 phyla, 118 families, and 232 genera were identified in the whole worm microbiota samples (Additional File 1: Table S1). The mean relative abundance of the five most abundant phyla are presented in Fig. 1A. There were no significant differences between groups for either alpha or beta diversity (Fig. 1B, C).

When comparing samples for shared genera, 28 were common among the four groups, and each group had at least 20 unique genera (Fig. 1D). A list of taxa found only within worms or found within worms with a numerically higher relative abundance compared to the horse is presented in Table 1. Only one, *Pelomonas*, had significantly different relative abundance based upon sex, with males having a significantly higher relative abundance than immatures (*P* = 0.0449), where the genus was not detected.

**Table 1:** Taxa found within more than one worm specimen, or within worms with a higher relative abundance (RA) in comparison to horse jejunum. The *P*-values represent statistical differences in RA between sexes within each classification

Classification	Sex	Prevalence	Mean RA	95% CI	*P*-value
		(%)	(%)	(%)	
*Bacillus*	I	37.50	0.82	(0.00–2.25)	0.4351
	M	28.57	0.17	(0.00–0.48)	
	F	12.50	0.04	(0.00–0.14)	
F: *Mycoplasmataceae*	F	50.00	0.09	(0.01–0.18)	0.6185
	M	28.57	0.21	(0.00–0.61)	
	I	25.00	0.07	(0.00–0.18)	
F: *Veillonellaceae*	F	25.00	0.16	(0.00–0.45)	0.7741
	M	14.29	0.02	(0.00–0.05)	
	I	12.50	0.31	(0.00–1.00)	
*Fusobacterium*	F	37.50	0.19	(0.00–0.44)	0.5902
	M	28.57	0.15	(0.00–0.39)	
	I	12.50	0.11	(0.00–0.35)	
*Janthinobacterium*	I	62.50	3.52	(0.00–7.66)	0.1552
	M	42.86	0.55	(0.00–1.50)	
	F	37.50	0.63	(0.00–1.39)	
*Nocardioides*	I	50.00	0.75	(0.00–1.57)	0.2517
	M	28.57	0.21	(0.00–0.52)	
	F	12.50	0.13	(0.00–0.41)	
*Pelomonas*	M	57.14	0.44	(0.04–0.84)	0.0379
	F	12.50	0.12	(0.00–0.38)	
*Sarcina*	M	100.00	22.13	(4.91–39.36)	0.3540
	H	100.00	9.29	(0.00–21.94)	
	F	100.00	27.94	(14.28–41.60)	
	I	87.50	25.37	(6.60–44.14)	

*I* immature, *M* male, *F* female, *H* horse

Only ANCOM-BC and DESeq2 returned any differentially abundant taxa for the whole worm samples, and none of them were shared. The DESeq2 results indicated that *Enterococcus* was differentially abundant across samples (*P* = 0.0058) and ANCOM-BC indicated that P: Proteobacteria (*P* = 0.0003) and *Sphingomonas* (*P* = 0.0003) were differentially abundant between female parasite and HJ samples.

### Adult *Parascaris* spp. gonad and intestinal microbiota

**Fig. 2:**
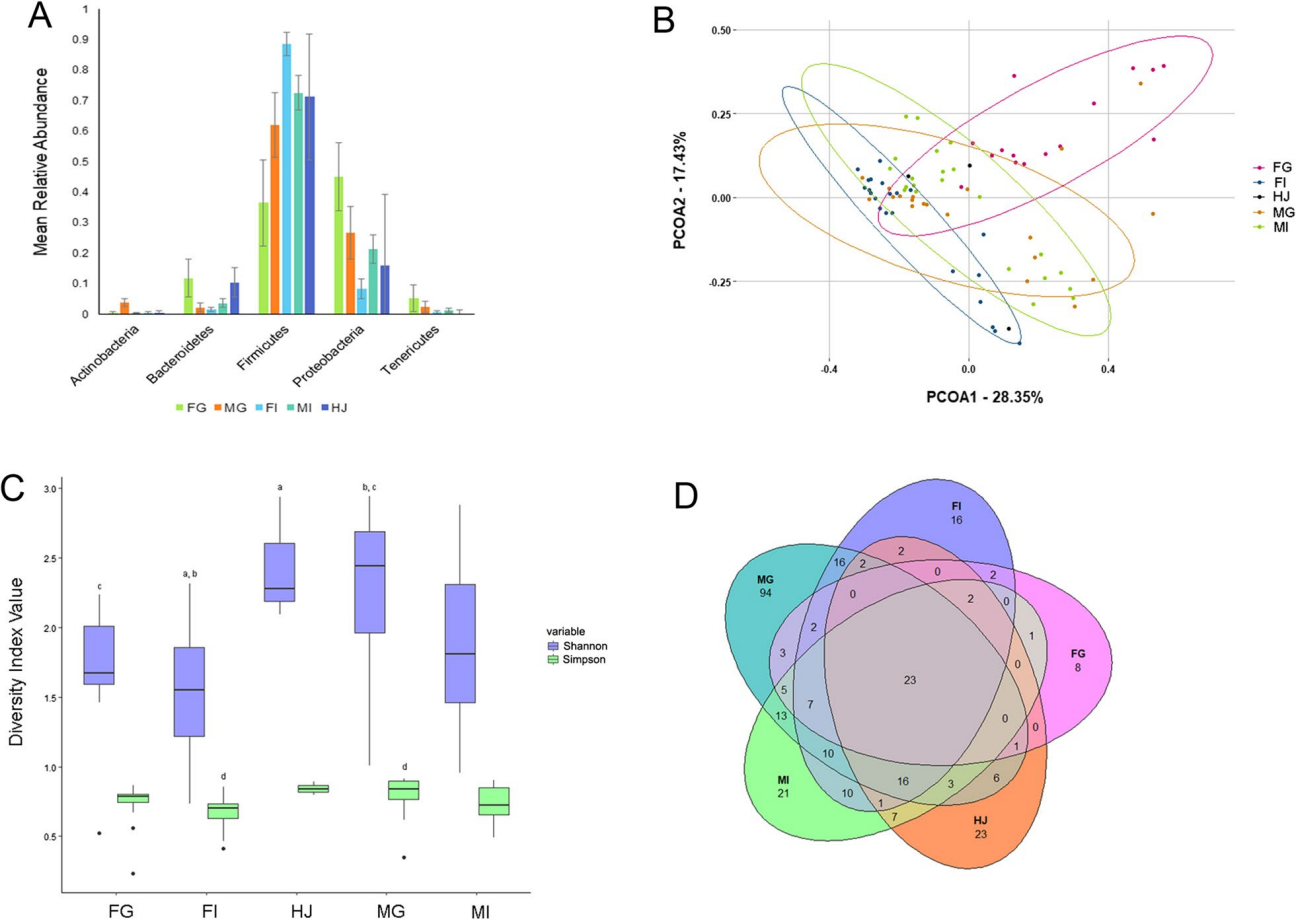
Diversity metrics, phylum relative abundance (RA), and shared genera for whole worm microbiota. **A** Mean RA of the five most abundant phyla, where error bars represent 95% confidence intervals. **B** Principal coordinate analysis plot based on Bray–Curtis dissimilarities. **C** Alpha diversity box plot showing both Shannon and Simpson alpha diversity, where • denotes outliers, and the same letters indicate significant differences. **D** Venn diagram showing number of shared genera between groups. *FG* female gonad, *FI* female intestine, *HJ* horse jejunum, *MG* male gonad, *MI* male intestine

Overall, a total of 22 phyla, 145 families, and 294 genera were identified in samples from this study (Additional File 2: Table S2). The mean relative abundance of the five most abundant phyla are presented in Fig. 2A. Alpha diversity was significantly different based upon both sex and organ (Fig. 2C). Simpson alpha diversity was significantly different between FI and MG (*P*= 0.0018). Shannon alpha diversity was significantly different between FG and MG (*P* = 0.0130), FI and HJ (*P* = 0.0383), and FI and MG (*P* = 0.0001) at the genus level. Beta diversity (Fig. 2B) was significantly different between MG and FG (*P* = 0.0006), MI and FG (*P* = 0.0093), and MG and FI (*P* = 0.0041). While not statistically significant based upon the alpha value set for this study, beta diversity tended to differ between MI and FI (*P* = 0.05602) and MG and HJ (*P* = 0.05776).

Comparison of all genera within groups indicated 23 shared genera across all groups and at least eight unique genera for each group (Fig. 2D). A summary of taxa found only within worms or found within worms with a higher relative abundance compared to the horse is presented in Table 2.

**Table 2:** Taxa found at prevalence of ≥ 50% within worms only, or with higher relative abundance compared to the horse

Classification	Location	Prevalence	Mean RA	95% CI
		(%)	(%)	(%)
*Aminobacter*	HJ	66.67	3.61	(3.50–3.71)
	MI	56.52	1.47	(1.42–1.52)
	FG	53.33	5.86	(5.41–6.30)
	FI	40.91	0.40	(0.32–0.49)
	MG	10.00	1.07	(0.00–2.47)
*Bacillus*	MG	65.00	1.01	(0.95–1.06)
	MI	30.43	0.15	(0.11–0.18)
	FI	18.18	0.10	(0.03–0.16)
F: *Mycoplasmataceae*	FG	86.67	1.23	(0.88–1.59)
	MI	78.26	0.67	(0.00–1.49)
	FI	72.73	0.16	(0.00–5.20)
	MG	15.00	0.09	(0.00–2.25)
*Gemella*	MI	60.87	0.59	(0.42–0.75)
	FI	59.09	0.51	(0.37–0.66)
	MG	25.00	0.24	(0.02–0.47)
	FG	6.67	0.05	(0.00–0.18)
*Janthinobacterium*	MG	85.00	5.02	(4.98–5.07)
	HJ	66.67	0.92	(0.75–1.09)
	MI	39.13	0.72	(0.66–0.77)
	FG	33.33	4.32	(4.24–4.40)
	FI	18.18	0.24	(0.16–0.32)
*Ralstonia*	MI	69.57	1.06	(1.05–1.07)
	FG	60.00	2.28	(2.27–2.30)
	MG	50.00	1.61	(1.60–1.63)
	HJ	33.33	0.11	(0.09–0.13)
	FI	22.73	0.14	(0.11–0.17)
*Reyranella*	HJ	100.00	3.01	(2.97–3.05)
	FG	93.33	12.16	(12.13–12.19)
	MI	78.26	5.23	(5.20–5.27)
	FI	59.09	0.90	(0.89–0.90)
	MG	15.00	1.21	(1.21–1.22)
*Sphingomonas*	MG	85.00	4.91	(4.84–4.98)
	MI	73.91	2.70	(2.65–2.75)
	HJ	66.67	1.93	(1.88–1.98)
	FG	60.00	8.67	(8.59–8.75)
	FI	36.36	0.37	(0.32–0.43)

*RA* relative abundance, *CI* confidence interval* FG* female gonad (*n* = 15), *FI* female intestine (*n* = 22), *HJ* horse jejunum (*n* = 3), *MG* male gonad (*n* = 20), *MI* male intestine (*n* = 23)

All genera had significant differences when Kruskal–Wallis tests were performed. A full table of *P*-values resulting from Dunn’s testing can be found in Table 3. All four differential abundance analysis methods returned statistically significant results for the organ data, and two were shared across all methods. *Sarcina* was differentially abundant across all four methods (*P*< 0.0001) and *Veillonella* was differentially abundant for DESeq2 and ANCOM-BC (*P* < 0.0001), corncob (*P*= 0.0008), and metagenomeSeq (*P* = 0.0118).

**Table 3:** Results of Dunn’s tests with Bonferroni correction for bacterial taxa found in *Parascaris* spp. organs, presented as *P*-values

	*Aminobacter*	*Bacillus*	F: *Mycoplasmataceae*	*Gemella*	*Janthinobacterium*	*Ralstonia*	*Reyranella*	*Sphingomonas*
FG–FI	0.3892	1.0000	0.0074	0.0155	0.5203	0.0084	< 0.0001	0.0017
FG–MG	0.0239	< 0.0001	< 0.0001	1.0000	0.0382	1.0000	< 0.0001	1.0000
FI–MG	1.0000	0.0010	0.2123	0.2486	< 0.0001	0.2722	1.0000	0.0002
FG–MI	1.0000	0.5265	0.3178	0.0069	1.0000	1.0000	0.1564	1.0000
FI–MI	0.6156	1.0000	0.8418	1.0000	1.0000	0.0153	0.0528	0.0251
MG–MI	0.0320	0.0163	0.0022	0.1315	0.0010	1.0000	0.0011	0.9760

*FG* female gonad, *FI* female intestine, *MG* male gonad, *MI* male intestine

### *Parascaris* spp. shared microbiota

**Fig. 3:**
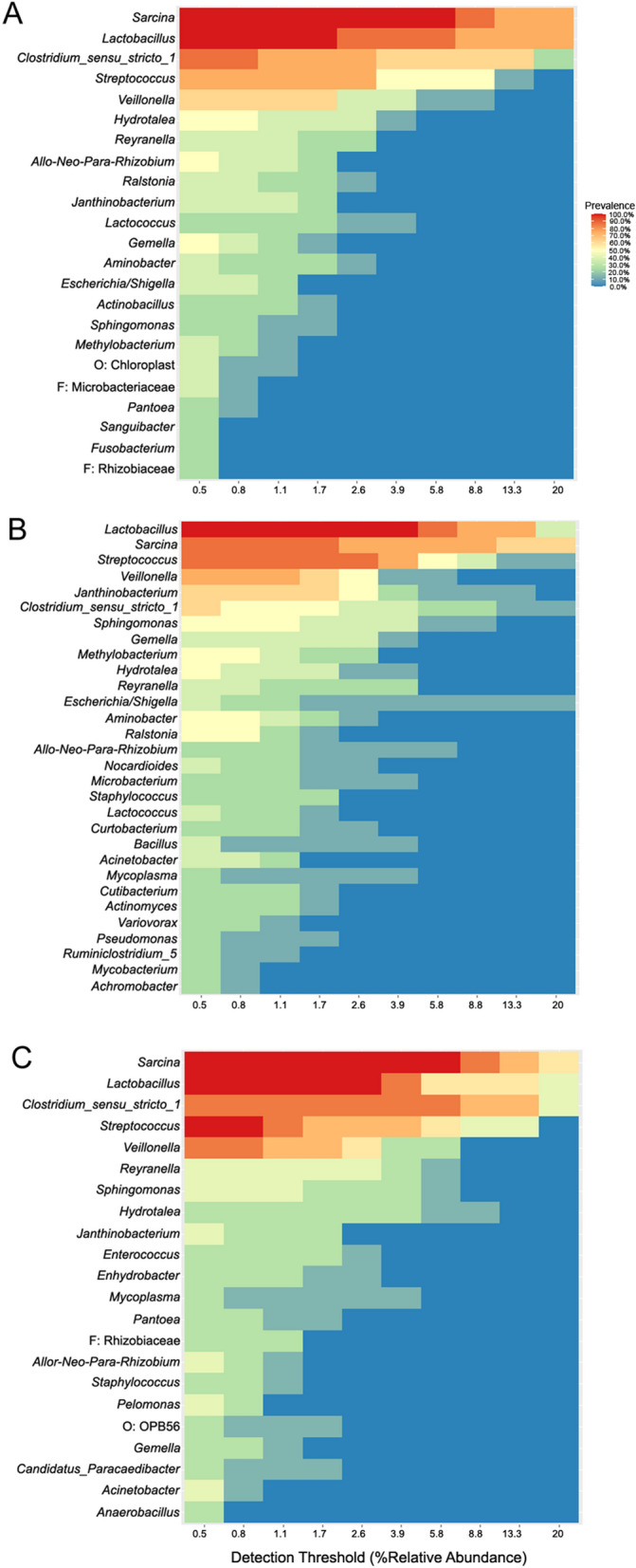
Shared microbiota heat plots showing genera with prevalence > 20% and relative abundance > 0.05% for **A** female parasites, **B** immature parasites, and **C** male parasites

**Fig. 4:**
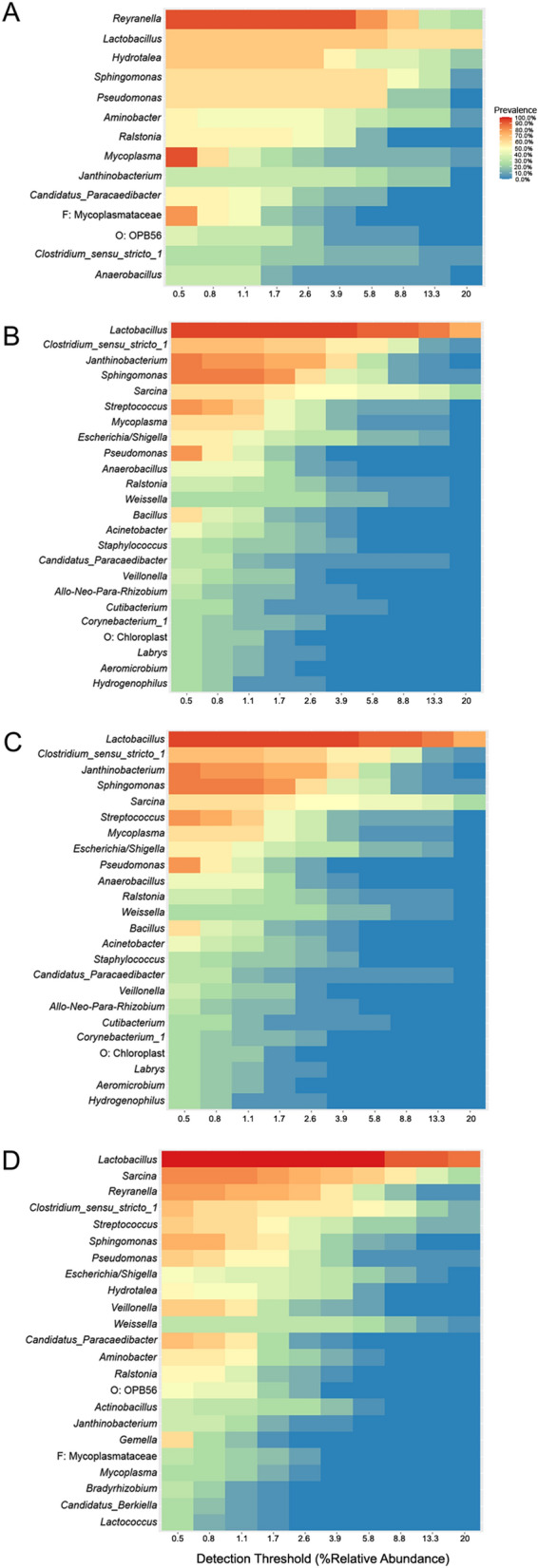
Shared microbiota heat plots showing genera with prevalence > 20% and relative abundance > 0.05% for **A** female gonad, **B** female intestine, **C** male gonad, and **D** male intestine

Heat plots generated to visualize the shared microbiota for the whole worm study are shown in Fig. 3. Comparison of these shared microbiota indicated that at least two taxa were unique to each group. Heat plots generated to visualize the shared microbiota for each group in the organ study are shown in Fig. 4. Comparison of these microbiota indicated unique taxa for MG and MI, and a total of four shared taxa across all groups.

Combined shared microbiota results from both whole worm and organ microbiota studies included 68 worms and identified 11 shared genera between male, female, and immature whole worm microbiota and combined male and female organ microbiota. The 11 genera were *Acinetobacter*, *Allorhizobium*-*Neorhizobium*-*Pararhizobium*-*Rhizobium* (ANPR), *Clostridium* *sensu stricto 1*, *Gemella*, *Janthinobacterium*, *Lactobacillus*, *Reyranella*, *Sarcina*, *Sphingomonas*, *Streptococcus*, and *Veillonella*. The prevalence and relative abundance data for each of these genera are presented in Table 4.

**Table 4:** Summary of mean percent prevalence, percent relative abundance (RA), and 95% confidence intervals (CI) for 11 genera shared among all groups with at least 20% prevalence

Genus	Whole worm				Organs			
	Location	Prevalence (%)	RA (%)	95% CI	Location	Prevalence (%)	RA (%)	95% CI
*Acinetobacter*	I	50.00	0.51	(0.00–1.01)	MG	45.00	0.89	(0.29–1.49)
	M	42.86	0.39	(0.01–0.76)	MI	26.09	0.07	(0.02–0.12)
	F	25.00	1.57	(0.00–4.95)	FI	18.18	0.05	(0.00–0.10)
					FG	13.33	0.12	(0.00–0.35)
ANPR	F	50.00	0.72	(0.07–1.38)	MG	40.00	0.53	(0.07–1.00)
	M	42.86	0.41	(0.00–0.94)	FI	27.27	0.25	(0.04–0.45)
	I	25.00	0.92	(0.00–2.60)	MI	26.09	0.14	(0.02–0.26)
					FG	13.33	0.12	(0.00–0.30)
*Clostridium* *sensu stricto 1*	F	87.50	14.66	(4.30–25.02)	FI	72.73	7.41	(2.66–12.16)
	M	85.71	17.78	(5.66–29.90)	MG	70.00	6.64	(3.87–9.41)
	I	62.50	6.13	(0.00–14.27)	MI	69.57	11.10	(4.25–17.95)
					FG	26.67	6.75	(0.00–18.75)
*Gemella*	F	50.00	0.66	(0.00–1.32)	MI	60.87	0.59	(0.34–0.83)
	I	37.50	1.32	(0.00–2.81)	FI	59.09	0.51	(0.25–0.77)
	M	28.57	0.36	(0.00–0.82)	MG	25.00	0.24	(0.01–0.48)
					FG	6.67	0.05	(0.00–0.15)
*Janthinobacterium*	I	62.50	3.52	(0.00–7.39)	MG	85.00	5.02	(3.16–6.89)
	M	42.86	0.55	(0.00–1.50)	MI	39.13	0.72	(0.16–1.27)
	F	37.50	0.63	(0.00–1.34)	FG	33.33	4.32	(0.49–8.16)
					FI	18.18	0.24	(0.00–0.54)
*Lactobacillus*	M	100.00	24.15	(9.03–39.28)	FI	100.00	41.34	(34.52–48.15)
	I	100.00	20.15	(9.26–31.03)	MI	100.00	36.06	(28.71–43.42)
	F	100.00	30.46	(17.43–43.50)	MG	95.00	32.31	(23.82–40.80)
					FG	66.67	25.82	(13.65–38.00)
*Reyranella*	I	50.00	1.53	(0.00–3.51)	FG	93.33	12.16	(8.05–16.27)
	M	42.86	2.12	(0.00–4.93)	MI	78.26	5.23	(2.83–7.63)
	F	37.50	1.00	(0.00–2.19)	FI	59.09	0.90	(0.50–1.30)
					MG	15.00	1.21	(0.00–2.89)
*Sarcina*	M	100.00	22.13	(6.18–38.08)	FI	90.91	26.03	(18.73–33.33)
	F	100.00	27.94	(15.16–40.72)	MI	82.61	13.82	(7.89–19.74)
	I	87.50	25.37	(7.81–42.92)	MG	60.00	10.05	(4.85–15.25)
					FG	20.00	1.33	(0.00–2.91)
*Sphingomonas*	I	50.00	2.61	(0.00–5.30)	MG	85.00	4.91	(2.60–7.22)
	M	42.86	2.39	(0.00–5.09)	MI	73.91	2.70	(1.48–3.92)
	F	25.00	0.37	(0.00–0.93)	FG	60.00	8.67	(3.70–13.64)
					FI	36.36	0.37	(0.13–0.62)
*Streptococcus*	M	100.00	10.05	(4.67–15.43)	MG	80.00	3.09	(1.05–5.13)
	I	87.50	9.16	(2.77–15.55)	FI	68.18	4.86	(1.42–8.31)
	F	75.00	6.34	(2.09–10.58)	MI	65.22	4.90	(1.82–7.98)
					FG	20.00	0.48	(0.00–1.13)
*Veillonella*	M	85.71	3.75	(1.70–5.79)	FI	81.82	3.06	(1.27–4.85)
	I	75.00	2.38	(0.83–3.93)	MI	69.57	1.82	(0.78–2.87)
	F	75.00	2.97	(0.14–5.80)	MG	50.00	0.58	(0.19–0.97)
					FG	13.33	0.21	(0.00–0.55)

*F* female, *I* immature, *M* male, *FI* female intestine, *FG* female gonad, *MI* male intestine, *MG* male gonad, *ANPR Allorhizobium*-*Neorhizobium*-*Pararhizobium*-*Rhizobium*

## Discussion

This study described the microbiota of a population of *Parascaris* spp. in a University of Kentucky parasitology research herd and compared microbiota of small intestinal life stages as well as different organs in adult parasites. This is the first study to describe the microbiota of an equine-specific parasite, an ascarid parasite, and individual nematode parasite organs. Mounting evidence indicates an important role in overall fitness of the parasite microbiota [63–68, 82, 84–86], and that a common core microbiota is maintained throughout the life-cycle and between sexes [73, 80]. The common core microbiota consists of taxa that are shared by all, or most, members of a given group [126, 127]. The prevalence and abundance of a given microbiota member are not necessarily tied to function, and rare taxa can be essential for host survival [128, 129]. The common core, however, does allow for an understanding of host-microbiota, phylogenetic, and population-level microbiota composition [130]. While the thresholds for shared microbiota were low for this study, at 20% prevalence, core microbiomes have been determined with such a low prevalence [80]; however, a more conservative approach was taken in this study, and the taxa in common are referred to as shared rather than core or common core microbiota.

Among the 11 shared genera identified across groups in this study, *Sarcina* and *Veillonella* were identified as differentially abundant in the organ microbiota study. All genera were identified in the horse jejunal microbiota, and all except *Reyranella* have previously been identified in equine gastrointestinal microbiota [131–137]. *Reyranella*, however, has previously been identified in other microbiota such as human neonatal and vaginal samples [138], fish [139–141] and shrimp [139]. *Reyranella* are gram-negative, non-motile, microaerophilic rod-shaped bacteria that have weak urase activity, oxidize CO to CO2, and are generally found in water and soil samples [142]. Reyranella is a relatively new genus, with the type species *R. massiliensis* first being described in 2011, and there are at present five named species within the genus [142]. *Reyranella*, along with *Acinetobacter* and *Sphingomonas*, has also been previously identified as a reagent contaminant [143, 144]; however, that is unlikely to be the case in this study, because three negative reagent controls were used and all ASVs found in the controls were removed from analysis, and the decontam [104] pipeline was also used, which takes into account sample concentration to remove contaminant sequences.

Comparisons between whole worm and organ results suggest that whole organism analysis of microbiota for helminths may mask nuances in microbiota structure within an organism. The organ study identified 27 more families and 62 more genera than were found in the whole worm study, including those that were unique to different sexes and organs. It also highlighted differences in relative abundance, such as the higher relative abundance of *Aminobacter*, F: *Mycoplasmataceae*, *Ralstonia*, and *Reyranella* in the FG and of *Sphingomonas* and *Janthinobacterium* in both MG and FG. These types of nuances that were not evident in the whole worm study are important because the microbiota can play an important role in parasite reproduction, as previously mentioned. Future studies establishing relative abundance trends in the global *Parascaris* spp. population, metabolomic and metaproteomic studies, and in vitro studies will be essential for determining the part that these genera play in the overall reproductive success of the parasite.

Out of the few helminth parasite microbiota studies completed to date, *H. contortus* and *Trichuris* spp. are the only other parasites of veterinary importance to have had their microbiota analyzed. Four microbiota studies have been completed for *H. contortus* [73–76] and two for *Trichuris* spp. [77, 78]. Three of those used a 16S rRNA hypervariable region and Illumina NGS to analyze the microbiota of different life stages as well as male and female *H. contortus* [73, 75] and a small number of *Trichuris* spp. [77]. Comparing microbiota studies with different methods is not always ideal because many factors such as the DNA extraction method [145, 146], PCR primers [147, 148], library preparation [149], and database [150, 151] can affect the results. One *H. contortus* [75] used a different DNA extraction method from that described herein but used the same database for taxonomic assignment and the same library preparation protocol, and thus the results can be compared with a good level of confidence. Another *Trichuris* spp. study used the same 16S rRNA hypervariable region and library preparation method as described herein, but different DNA extraction and taxonomic database, and so the results must be compared with more caution.

In one *H. contortus* study [75], male specimens ha higher alpha diversity than females, but both sexes clustered together for beta diversity. This is similar to results found in the present study, where there were no discernible clusters specifically for male or female worms for beta diversity. Alpha diversity was similar for the whole worm study; however, male *Parascaris* spp. gonad and intestine both had higher alpha diversity than female organs when parsed out. Out of the most prevalent genera in the *H. contortus* study, only *Acinetobacter* was shared with the *Parascaris* spp. shared microbiota, and out of the unique genera found in either male or female *H. contortus*, only *Corynebacterium* 1 and *Prevotella* were shared between the two parasite species. They were found in the MG, FI, and MI, and in the MG, respectively, both with prevalence ≤ 25.0%.

The *Trichuris* spp. study mainly contained samples of parasite intestine and a total of only seven specimens, three males and four females [77]. The genera *Acinetobacter* and *Sphingomonas* were the only two *Parascaris* spp. shared microbiota that were also found in *Trichuris* spp. [77]. Shannon alpha diversity did not show any differences between the samples; however, the sample size for this study was small, and no comparisons were made between sexes or the two species—*T. trichiura* and *T. suis*—examined in this study [77].

The five most abundant phyla for both whole worm and organ microbiota studies in *Parascaris* spp. were Firmicutes, Proteobacteria, Bacteroidetes, Actinobacteria, and Tenericutes (Figs. 1A, 2A). Firmicutes was the most prevalent phylum for all groups in both *Parascaris* spp. studies; however, this differs from results found in the *H. contortus* and *Trichuris* spp. studies, where Proteobacteria was the most prevalent phylum [73, 75, 77]. In another *Trichuris* spp. study, the phylum Bacteroidetes was the most prevalent [78]. In three *H. contortus* studies, a limited number of phyla were detected overall [73, 74, 76], whereas 23 were identified in another [75], which is similar in number to the 22 phyla identified in this study. A total of 36 phyla were identified in *Trichuris* spp. [77], over 50% more than found in *Parascaris* spp. Overall, there were some similarities between the *Parascaris* spp., *Trichuris* spp., and *H. contortus* microbiota despite some differences in methodology. It is important to note, however, that there are substantial biological differences among all three parasite types. *Parascaris* spp. reside in the small intestine, feed on intestinal content, have migratory stages throughout the host [7], and are a clade III nematode [152]; *H. contortus* resides in the abomasum, feeds upon blood, does not have migratory stages in the host [153], and belongs to nematode clade V [152]; and *Trichuris* spp. reside in the caecum, burrow into the mucosal epithelium, do not have migratory stages in the host [154], and belong to nematode clade I [152]. These biological differences could have a substantial effect on microbiota composition due to differences in hosts, genetics [155, 156], diet [157], and environmental exposure throughout the life-cycle [158, 159].

Further studies assessing microbiota function and localization will be necessary to determine whether some of these bacterial genera are important for parasite fitness, what role they play in parasite biology, and whether they are passed down via vertical transmission or acquired from the environment. This study was limited by small sample sizes, both in number of worms and number of foals represented. A total number of six foals were represented in the entirety of the study, which was of course limited by the number of foals with a parasite burden at the time of necropsy. Jejunum content from these foals, however, does represent every population from which worms were collected, and while the number of reads (range 4023–10,431) may at first appear low, the number of ASVs (769) and genera (136) is similar to findings in previous studies. The jejunum tends to have lower bacterial diversity than other gastrointestinal compartments, and previous studies have identified 293 OTUs in the gastrointestinal tract [160], 209 OTUs in the small intestine [161], and 500 unique sequences and 135 OTUs in the jejunum [162]. Additionally, the number of parasites, particularly for the whole worm study where fewer than 10 individuals were available for each group, is another limitation, and results may change with larger sample sizes. However, when comparing results from the whole worm and organ studies, dissecting *Parascaris* spp. may provide more nuance and insight into the parasite microbiome and thus may be a better way forward when studying the microbiome of this particular organism.

While is it not possible to discern any functional implications of microbiota from the present research, some of the members may be worth further investigation. *Reyranella*, for example, has been previously identified in the human vaginal microbiota and was also identified in this study at a higher prevalence and relative abundance in the FG compared to the other investigated sites, suggesting a possible function in the female reproductive tract. Additionally, the two differentially abundant genera, *Sarcina* and *Veillonella*, are also worth deeper investigation as a differentiating factor between different organ compartments of *Parascaris* spp. Future research will provide further insight into the possibility of using microbiota manipulation for the control of *Parascaris* spp. and development of new anthelmintic treatments.

## Conclusions

This study is the first to characterize the microbiota of an ascarid parasite, a parasite affecting horses, and the first parasitic nematode microbiota to include analysis of separate organs. A group of shared microbiota for the study population was determined to consist of 11 bacterial genera, two of which—*Sarcina* and *Veillonella*—were differentially abundant in organs. The gonad and intestine of female and male specimens were found to have differences for both alpha and beta diversity, suggesting that there are potentially important nuances in organ compartment microbiota versus whole-organism microbiota studies for parasitic nematodes. Ultimately, more research is needed with larger sample sizes and diverse populations to parse out differences in microbiota of *Parascaris* spp., and functional studies are needed to determine what role the parasite microbiota plays in host fitness.

## Supplementary Information

**Additional file 1: Table S1.** Relative abundances of all genera, families, and phyla for each whole worm sample.

**Additional file 2: Table S2.** Relative abundances of all genera, families, and phyla for each organ sample.

## Data Availability

Sequencing data were submitted to the National Center for Biomedical Information (NCBI) Sequence Read Archive under accession number PRJNA851371; BioSample accession numbers SAMN29223031–SAMN29223159.

## Abbreviations

FG: Female gonad
FI: Female intestine
HJ: Horse jejunum
MG: Male gonad
MI: Male intestine
